# Dentists in action: a profession on-screen (1913-2013)

**DOI:** 10.1038/s41415-022-4145-6

**Published:** 2022-05-27

**Authors:** Samuel Gierok, Shahzeb A. Mirza, Axel Karenberg

**Affiliations:** Institute for the History of Medicine and Medical Ethics, University Hospital Cologne, Germany

## Abstract

**Background** Fictional portrayals of dentists in feature films have remained largely unexamined to date. The aim of this review is consequently to catalogue and analyse available films produced by US entertainment industry that present 'dentists in action'.

**Methods** Relevant motion pictures were identified by means of keyword-based inquiries in search engines, online databases, websites and by handsearch.

**Results** Between 1913-2013, almost 60 American films with dental treatment as a motif were released. Dentists on-screen appeared mainly in comedies and mostly as supporting actors. Surgical treatments dominated in earlier films and tooth-preserving therapy in later films; other fields of dentistry were marginalised. The time lag between a dental innovation and its screen debut varied between 50 years (x-rays) and 10 years (turbine). For a long time, filmmakers refused to allow female dentists to appear on screen. Although there is no consistent stereotype of a dentist, the figure of 'Dr Awkward' can be attributed to the silent film era, 'Dr Prosperous' to the 1960s/1970s and 'Dr Evil' to the 1980s/1990s.

**Discussion** Popular media does not only reflect aspects of reality; they also create reality and establish a professional image. Thus, filmic representations of dentists have an immediate effect on an audience of millions of movie-goers and television viewers. Greater attention should be devoted to the interplay of cinematic and dental art by both dental professionals and film historians.

## Key points

- Provides examples of how twentieth-century filmmakers portrayed practising dentists.
- Enhances understanding of how film trends and dental innovations shaped the image of the profession.
- Makes us familiar with popular Hollywood stars acting as dentists on-screen.

## Background

'I think that I have just viewed the ultimate in the downgrading of a profession'. With these words, the author of a letter to the editor of a dental journal in 1976 condemned the appearance of a torturing dentist with a Nazi past in the notorious US film *Marathon man*. At the same time, he called on the American Dental Association (ADA) to establish a 'watchdog committee over the entertainment industry and the media'.^1^ But does the former Nazi henchman really embody the typical doctor of dental surgery on-screen? And how does dentistry at large look through the lens of filmmakers? Neither enthusiasts of dental history nor film scholars have systematically investigated repercussions of dental practice in films. After all, several productions have been briefly mentioned in this journal.^2^

This overview attempts to catalogue and analyse American feature films that show a dental treatment. The following topics are in focus: how many such motion pictures do exist and to which film genres can they be assigned? Which directors took up the motif? Which well-known actors and actresses impersonated dentists? How did technical innovations and cinematic trends affect the films? What 'image' of male and female dentists have producers and scriptwriters created over the decades? These questions will be examined in this paper.

## Materials and methods

A keyword-based screening in search engines (Google), online databases (Moviepilot.de; IMDB) and websites (https://www.drschminke.de/filme.html) provided a first overview. After chronological sorting, all available productions were carefully viewed and evaluated.

The following survey is limited to feature films from the period 1913-2013 that were (co-) produced in the USA. Furthermore, only films depicting a dental character, a patient and a dental examination and/or treatment were included. Documentaries, as well as educational and pure children's films, series' and productions for television were excluded. For this reason, the widely-known episode from the TV series *Columbo* titled *Uneasy lies the crown* (1990) could unfortunately not be included. In this episode, a dentist commits murder by means of a poisoned dental filling. Despite thorough research work, it must be assumed that some works have been overlooked.

## Results

Based on these criteria, almost 60 productions have been included in this study. The period under investigation has been divided into five sections, each covering about 20 years. One film example from each of these periods is discussed in detail and the rest of these films are presented in tabular form. Summaries of filmic trends and dental developments place the presented films in a larger context.

### 1913-1932: tooth extractions with and without anaesthetisation

The visit to the dentist was already one of the favourite subjects in silent films (Table 1). Charlie Chaplin (1889-1977), Snub Pollard (1889-1962) and the duo of Stan Laurel (1890-1965) and Oliver Hardy (1892-1957) were the most famous comedians to be seen on their brief adventures in a dental practice. As the film titles *Laughing gas* (1-2), *The dippy dentist* (1-3) and *Leave 'em laughing* (1-7) already suggest, a 'Dr Awkward' often triggered a slapstick cascade through a failed anaesthetic. Dramaturgically, an extraction with ineffective or no anaesthetisation dominated, which can be explained, above all, by the intended effect: in the 'cinema of laughter', sensation-hungry spectators feasted on the fate of afflicted fellow human beings and entertained themselves excellently thanks to crude situation comedy. Against this background, technical innovations such as intraoral x-ray examinations, nerve block anaesthesia and cast crowns received only rare and at most marginal attention (1-4, 1-5). In the 'cinema of narrative', however, the dentist's role slowly developed into a component of a longer plot (1-5). With the advent of the sound film around 1930,^3^ a fictional specialist could even be at the centre of an imaginary story.

**Table 1:** Films portraying dentists in action (1913-1932)

No.	Title	Country	Year	Director
1-1	*The tramp dentist (lost)*	USA	1913	Allen Curtis
1-2	*Laughing gas*	USA	1914	Charles Chaplin
1-3	*The dippy dentist*	USA	1920	Alfred J. Goulding
1-4	*Tommy Tucker's tooth*	USA	1922	Walt Disney
1-5	*Greed*	USA	1924	Erich von Stroheim
1-6	*Clara cleans her teeth*	USA	1927	Walt Disney
1-7	*Leave 'em laughing*	USA	1928	Clyde Bruckman
1-8	*Pardon us*	USA	1931	James Parrot
1-9	*The dentist*	USA	1932	Leslie Pearce

Still committed to slapstick, the popular comedian W. C. Fields (1880-1946) starred in the short *The dentist* (1-9). This film set trends that influenced the next decades: comedy as the character's 'natural biotope'; contemporary practice interiors with dentist's chairs, a spittoon, an electric drill and Doriot's transmissions; a more-or-less detailed rendering of treatment procedures; the appearance of a young female assistant in a white bonnet; and not to forget the cliché of an expert who has made it with his profession and can spend his free time on the golf course.

### 1933-1956: dental heroes in focus

In the years following the Great Depression, Hollywood's Golden Age began. Up to 100 films a year were produced by each of the major studios, from Universal and Metro-Goldwyn-Mayer to Warner Brothers and Twentieth Century Fox.^4^ During the same period, the number of dentists also grew: By 1930, the ADA had registered 36,000 members, who served about 85% of the market by the late 1940s.^5^ However, due to World War II, dentistry made only hesitant progress. After 1945, the further development of intraoral x-ray technology to extraoral pantomography was successful.^6^ Dental technology experienced a certain upswing with new polymer resin and prosthodontic impression materials and for the public health sector, the introduction of nylon toothbrushes as well as water and toothpaste fluoridation played an important role.^7^

Practically, none of it found its way onto the screen. In general, 'celluloid dentists' rarely appeared in these years; their professional activity became visible as a short episode at best (Table 2). *One Sunday afternoon* starring Cary Grant (1904-1986) (2-2) tells the story of a small-town doctor disappointed in life, who seeks revenge on a rich boyhood friend. More successful was a remake (2-8) directed by Raoul Walsh (1887-1980). In both productions, the treatment took up between 6-14 minutes in a running time of 90 minutes, a significantly larger share than in most earlier or later films. Conservative dentistry and extractions were now on an equal footing and for the first time, even prosthesis insertion could be seen. A second remake was set in 1890s New York (2-5). Just like a Western comedy starring Bob Hope (1903-2003), alias 'Dr Painless' (2-7), it offered viewers a glimpse into the past of US dentistry - or at least a cinema version of it.

**Table 2:** Films portraying dentists in action (1933-1956)

No.	Title	Country	Year	Director
2-1	*The merry old soul*	USA	1933	Walter Lantz
2-2	*One Sunday afternoon*	USA	1933	Stephen Roberts
2-3	*Buddy the dentist*	USA	1934	Ben Hardaway
2-4	*The awful tooth*	USA	1938	Nate Watt
2-5	*Strawberry blonde (first remake of 2-1)*	USA	1941	Raoul Walsh
2-6	*The great moment*	USA	1944	Preston Sturges
2-7	*The paleface*	USA	1948	Norman Z. McLeod
2-8	*One Sunday afternoon (second remake of 2-1)*	USA	1948	Raoul Walsh
2-9	*The noose hangs high*	USA	1948	Charles Baron

The biopic of William Thomas Green Morton (1819-1868) was planned to be a hymn to a true dental hero. After all, he had publicly demonstrated inhalation anaesthesia using sulphur ether barely 100 years earlier, giving young America its first important discovery to the medical world.^8^ The originally favoured film titles *Immortal secret*, *Great without glory*, *Morton the magnificent* and - based on the 1940 book by R. Fülöp-Miller - *Triumph over pain,* stressed this intention of Paramount Pictures. In contrast, director Preston Sturges (1898-1959) wanted to emphasise the serendipity of discovery, highlight the refractions in the hero's character and spice up the conventional triumph-and-tragedy drama with a good dose of humour. All this led to his dismissal and the somewhat lukewarm title *The* *great moment* (2-6). The story is told in flashbacks from the perspective of Morton's ageing widow and 'Dr Famous' can only be perceived as a practising dentist in two scenes, one during the visual inspection of a tooth status including palpatoric examination and the other as an excited spectator during an extraction performed using 'his' new anaesthetic. Overall, the largely authentic biography could convince neither viewers nor critics: the film flopped. Today this could be different, as a colleague recently judged: '*The* *great moment* may now be due for a general re-evaluation by film historians and critics who, like most folks, have never felt much affection for dentists past and present'.^9^

### 1957-1979: from filling to torture

At the beginning of the 1950s, television began to compete with cinema and the classic Hollywood era came to an end. In dentistry, on the other hand, a new era was dawning. With the pneumatically driven dental turbine, which was introduced in 1957, the preparation of cavities was much faster and more gentle on the pulp than before.^7^ The adhesive technique made dental sealants possible and was the basis for the insertion of composite fillings. The disposable syringe made of plastic simplified nerve block anaesthesia.^10^ Individual filmmakers admired the technical progress and even more the fact that, from now on, a female assistant was indispensable at the dental chair of a modern practice (Table 3). In addition to the dental unit and many accessories, x-ray machines and images - almost 50 years after their introduction into everyday diagnostics - were also represented on the set. These were extensively used by Dr Winston, alias Oscar-winner Walter Matthau (1920-2000), who marries his pretty assistant Stephanie, alias Oscar-winner Ingrid Bergman (1915-1982), at the end of the love story *Cactus flower* (3-4). Nevertheless, dentists remained a rarity in the cinema of the time. Screenwriters had little need for such a character throughout the 1960s. Even during the 'New Hollywood era' until the late 1970s, doctors were largely granted a minor supporting role in horror flicks (3-1), comedies (3-2, 3-8), Western parodies (3-3, 3-5) and romances (3-4).

**Table 3:** Films portraying dentists in action (1957-1979)

No.	Title	Country	Year	Director
3-1	*The little shop of horrors*	USA	1960	Roger Norman
3-2	*Kiss me, stupid*	USA	1964	Billy Wilder
3-3	*The shakiest gun in the west*	USA	1968	Alan Rafkin
3-4	*Cactus flower*	USA	1969	Gene Saks
3-5	*From noon till three*	USA	1975	Frank D. Gilroy
3-6	*Marathon man*	USA	1976	John Schlesinger
3-7	*The in-laws*	USA	1979	Arthur Hiller
3-8	*10*	USA	1979	Blake Edwards

A mainstream dentist on-screen was offered to the audience at the end of this era by Warner Bros. *The in-laws* (3-7), 'one of the funniest comedies of the year',^11^ ties a New York colleague of stiff and obliging manners in his prime (Alan Arkin) to a shady businessman (Peter Falk) and leads the duo against their will through a turbulent plot. Politically conservative, dentally progressive and economically successful, this is how this 'Dr Prosperous' of the late 1970s could best be characterised. As he shows his daughter's future father-in-law around the spacious practice, the latter remarks dryly that it 'looks like an absolute goldmine!' And indeed, nothing is lacking in the professional realm, from the modern unit with OT-lamp and a tray loaded with a pack of instruments to the OPG in a separate x-ray room - everything is mustered by the props department. Two detailed film sequences also illustrate that tooth preservation and prosthetic care have long since replaced extraction as the dominant motif. Against this backdrop, it becomes clear that director John Schlesinger (1926-2003) completely broke with tradition by introducing the torture dentist Dr Szell (Laurence Olivier). Nevertheless, the thriller *Marathon man* (3-8) drew crowds to the cinema: 'Dr Evil' opened up a new dramaturgical perspective for the dentist character and offered audiences a thrill that still keeps aficionados on the edge of their seats today.

### 1980-1999: the first female dentist on-screen

For dentists and film fans alike, the 1980s opened up new technical options. Thanks to the proliferation of VCRs, cineastes, as well as average consumers, could enjoy their favourite films in the comfort of their own homes. This boosted the production of feature film series' and caused film industry budgets to soar to unimagined heights.^12^ Almost revolutionary innovations also occurred at the dental chair: computer-aided design and manufacturing technology made it possible to have restorations produced by computer-controlled milling machines after scanning the findings in the patient's mouth.^13^ After decades of research into 'osseointegration', the era of implantology dawned, becoming the fastest growing sub-discipline of dentistry, which also found an early cinematic but slightly alienated adaptation in the comedy *Go for it* (4-1) starring Terence Hill (*1939) and Bud Spencer (1929-2016). At about the same time, hepatitis and AIDS infections increased the demands on practice hygiene: gloves, face masks and protective glasses had been standard since the mid-1980s^14^ and gradually found their way onto the screen (Table 4).

**Table 4:** Films portraying dentists in action (1980-1999)

No.	Title	Country	Year	Director
4-1	*Go for it*	USA/Italy	1983	Enzo Barboni
4-2	*Compromising positions*	USA	1985	Frank Perry
4-3	*Little shop of horrors (remake of 3-1)*	USA	1986	Frank Oz
4-4	*Burglar*	USA/Canada	1987	Hugh Wilson
4-5	*Honeymoon in Vegas*	USA	1992	Andrew Bergmann
4-6	*Serial mom*	USA	1994	John Waters
4-7	*Problem child 3*	USA	1995	Greg Beemann
4-8	*Houseguest*	USA	1995	Randall Miller
4-9	*The dentist*	USA	1996	Brian Yuzna
4-10	*Looking for Lola*	USA	1997	Boaz Davidson
4-11	*The dentist 2*	USA	1988	Brian Yuzna
4-12	*Lethal weapon 4*	USA	1998	Richard Donner

As in the 1930s, dentists - though now in increasing numbers - were to be found mainly in supporting roles in comedies of all kinds (4-2, 4-3, 4-5, 4-6, 4-7, 4-8, 4-10). At the same time, following the example of *Marathon man*, the subgenre 'dental horror' was cultivated: The sadistic main character in *The dentist* (4-9) and its sequel (4-11) also served the audience's worst fears and most bloodthirsty fantasies. In both machinations, director Brian Yuzna (*1949) simultaneously endeavoured to demonstrate scientifically-established standards in an almost documentary-like manner, from a focus on prophylaxis and radiation protection to implants and aesthetic dentistry (small wonder, a dental consultant was involved in both films).

This period also offers a first-rate cultural-historical innovation: the first appearance of a practising female dentist in a US feature film - some 120 years after the first woman graduated from a US dental college^15^ and almost 75 years after the profession became popular in silent films. The simple action film *Burglar* (4-4) increased the surprise effect by including a male assistant. After superficial anaesthetisation of the mucosa, Dr Cynthia Sheldrake (Lesley Ann Warren) places a block anaesthetic in the left mandible and then prepares a cavity. One can still see that her patient's (Whoopi Goldberg) teeth have a number of amalgam fillings. Even if examination and treatment were, as so often, only an insignificant subplot, the short scene forms a milestone in American film history.

### 2000-2013: dentists conquer the screen

In the new millennium, the trend towards high-budget productions continued. At the same time, filmmaking was popularised by means of the new possibilities offered by personal computers and the internet. Dentistry also benefited from advances in science, technology, materials research and various other fields. Digital x-ray, diagnostic imaging, intraoral cameras and scanners, CEREC technology, all-ceramic restorations, aligner therapy and a multitude of other innovations have changed everyday practice and have been perceived by the profession as 'key breakthroughs'.^16^ At the same time, the American public's perception of beauty was changing. Cosmetic dentistry and a 'Hollywood smile' were no longer a luxury for the few, but were considered standard of care by some dentists.

As a consequence of the increasing numbers of films and the growing relevance of dentistry, the character of the dentist became as popular as it had never been before. At least 17 such characters can be verified in almost one decade (Table 5). First since the silent film era, the film titles featured dentists and obviously aroused positive associations (5-1, 5-3, 5-16). Moreover, it was also the first time that an African American dentist (5-11) and a dentist couple (5-3) acted in a film, illustrating a social reality existing for decades.

**Table 5:** Films portraying dentists in action (2000-2013)

No.	Title	Country	Year	Director
5-1	*Novocaine*	USA	2001	David Atkins
5-2	*Pearl Harbor*	USA	2001	Michael Bay
5-3	*The secret lives of dentists*	USA	2002	Alan Rudolph
5-4	*Snow dogs*	USA	2002	Brian Levant
5-5	*Final destination 2*	USA	2003	David R. Ellis
5-6	*Finding Nemo*	USA	2003	Andrew Stanton
5-7	*The whole ten yards*	USA	2004	Howard Deutch
5-8	*Thumbsucker*	USA	2005	Mike Mills
5-9	*Charlie and the chocolate factory*	USA, UK, Australia	2005	Tim Burton
5-10	*Good luck Chuck*	USA/Canada	2007	Mark Helfrich
5-11	*Reign over me*	USA	2007	Mike Binder
5-12	*Wild hogs*	USA	2007	Walt Becker
5-13	*Ghost town*	USA	2008	David Koepp
5-14	*The hangover: part II*	USA	2011	Todd Phillips
5-15	*Horrible bosses*	USA	2011	Seth Gordon
5-16	*Spirit of a denture*	USA	2012	Alan Shelley
5-17	*Touchy feely*	USA	2013	Lynn Shelton

An Indian dentist appears in the 2008 romantic comedy *Ghost town* (5-13), but it is not he who plays the leading role, but his British colleague, Dr Pincus (Ricky Gervais), who has ended up in Manhattan. Viewers of this love story, spiced with a good dash of humour and sarcasm, are treated to panoramic slices of jaws with implant restorations and dental cleanings, as well as a flawless impression of the upper jaw and several filling therapies. But why did the main character have to be a dentist? It is possible that he was intended to be portrayed as particularly unsympathetic at the beginning of the plot and the filmmakers hoped to best achieve this effect by associating him with a visit to the dentist.

## Discussion and conclusions

Cinematic reflexes of medicine as a whole,^17^^,^^18^ as well as of individual specialties,^19^^,^^20^ have been studied extensively. However, apart from an overview of German cinema,^21^ the many links between the art of film and the art of dentistry have not yet received attention. This neglect is also due to methodical problems, since films with a dentist motif are hardly searchable systematically. Identification, review and interpretation of the heterogeneous material require an interdisciplinary approach. Therefore, the following ten conclusions are merely an attempt to lay the first stepping stones into hitherto uncharted territory. They are based on the analysis of US cinema presented above and cannot necessarily be applied to other countries with different cultural backgrounds:

1. Chronology and frequency: in a leading medium of the twentieth century, the dentist's role has shown a continuing presence from the very beginning. Until 1980, one dentist appeared on screen on average every three years; from 1980-2000 every three years. In the new millennium, this rate rose to more than one character per year. Similar figures can be demonstrated for German cinema^21^
2. Directors and actors: Hollywood greats were/are not rare behind the camera. Blake Edwards, Raoul Walsh, Billy Wilder and John Schlesinger are among the best known. Until 2000, Charlie Chaplin, W. C. Fields, Cary Grant, Bob Hope, Walter Matthau and Laurence Olivier, among others, portrayed a dentist; after that was Steve Martin, Keanu Reeves, Jennifer Aniston, Christoph Waltz and Kevin Spacey
3. Genre: filmic colleagues appear in almost all genres, from westerns to action and fantasy to thrillers. More than a half, however, appeared in comedies. By the 1960s at the latest, dental treatment had ceased to be an amusing element. This observation also applies to German productions^21^
4. Cast: the dentist rarely acted as the protagonist and was often a supporting actor - just as diagnostics and therapy almost always formed an episode lasting only a few minutes. However, the character has become so popular in the last 20 years that one could almost speak of one of the most important supporting roles in recent US film history
5. Dramatic composition: depending on the genre, the intended effect of a treatment scene could range from amusement to the creation of diffuse fear to true moments of terror. Some scriptwriters also used such sequences to symbolise the social advancement of a dentist or the patients' desires for aesthetic perfection
6. Authenticity I: feature films are not documentaries. In not a single work of fiction after 1945 would one find a complete treatment - only fragments. Directors followed the basic principles of their guild: dramatising, emotionalising, popularising and fragmenting.^22^ Especially in early film, cinematic codes developed, that is, the extraction of the wrong tooth or the demonstrative holding up of a tooth that has just been pulled. Later productions, on the other hand, often aimed to reproduce dental procedures as accurately as possible
7. Authenticity II: the form of therapy staged was more determined by cinematic possibilities than dental reality. Surgical and conservative options dominated. Prosthetics, prophylaxis and orthodontics were rarities and maxillofacial surgery did not feature at all. Apart from the silent era and historical narratives, the detailed equipment on set often reflected contemporary practice, depending on the budget
8. Authenticity III: the latency period that it took from a dental invention to its screen debut varied considerably. While x-ray technology developed over 50 years and nerve block anaesthesia around 40 years, turbine and contra-angle handpieces could be seen on-screen just over 10 years after their introduction. Conversely, productions from recent years presented a wealth of technical innovations in an almost semi-documentary manner
9. Feminisation: although the presence of female dentists in real life has steadily increased, producers and scriptwriters have long denied female dentists a cinema presence. This film character made its debut only at the end of the 1980s but you can count the respective roles on one hand up to the 2010s, with around 50% female dental students and 30% female dentists.^23^ German feature films took professional emancipation into account earlier and more extensively^21^
10. Image: there is no such thing as 'the' stereotype of the film dentist. Even within one and the same decade, the figure went through a variety of 'adaptations'. If one accepts terrible simplifications, 'Dr Awkward' can be ascribed to the silent film era and 'Dr Prosperous' to the 1960s/1970s. 'Dr Evil', released in the 1980s, was rather hesitantly emulated, even without a 'watchdog committee'.

Similar to literary^24^ and pictorial^25^ portraits, cinematic portrayals of dentists are a significant archive of everyday life. Within their boundaries, they capture scientific and technical developments and reflect interpersonal interactions and social assessments. This world, at the interface of reality and fiction, which fascinates millions of people, henceforth deserves greater attention with regard to 'dentists in action'.
